# Structural Modification of Nanomicelles through Phosphatidylcholine: The Enhanced Drug-Loading Capacity and Anticancer Activity of Celecoxib-Casein Nanoparticles for the Intravenous Delivery of Celecoxib

**DOI:** 10.3390/nano10030451

**Published:** 2020-03-02

**Authors:** Liuli Xv, Xinxin Qian, Yan Wang, Chenghuan Yu, Dingkui Qin, Yahui Zhang, Peng Jin, Qizhen Du

**Affiliations:** 1 The Key Laboratory for Quality Improvement of Agricultural Products of Zhejiang Province, College of Agricultural and Food Sciences, Zhejiang A & F University, Hangzhou 311300, China; xll2527@163.com (L.X.); xinxinqian@126.com (X.Q.); Yanwang@126.com (Y.W.); celeseric@hotmail.com (D.Q.); 9zyh@tongji.edu.cn (Y.Z.); 2 Experimental Animal Center of the Zhejiang Academy of Medical Sciences, Hangzhou 310013, China; Chyu@163.com

**Keywords:** celecoxib, delivery, casein nanoparticles, stability, anticancer

## Abstract

This study aims to stabilize loaded celecoxib (CX) by modifying the structure of casein nanoparticles through phosphatidylcholine. The results show that Egg yolk phosphatidylcholine PC_98T_ (PC) significantly increased the stability of CX-PC-casein nanoparticles (NPs) (192.6 nm) from 5 min (CX-β-casein-NPs) to 2.5 h at 37 °C. In addition, the resuspended freeze-dried NPs (202.4 nm) remained stable for 2.5 h. Scanning electron microscopy indicated that PC may block the micropore structures in nanoparticles by ultrasonic treatment and hence improve the physicochemical stability of CX-PC-casein-NPs. The stability of the NPs was positively correlated with their inhibiting ability for human malignant melanoma A375 cells. The structural modification of CX-PC-casein-NPs resulted in an increased intracellular uptake of CX by 2.4 times than that of the unmodified ones. The pharmacokinetic study showed that the Area Under Curve (AUC) of the CX-PC-casein-NPs was 2.9-fold higher in rats than that of the original casein nanoparticles. When CX-PC-casein-NPs were intravenously administrated to mice implanted with A375 tumors (CX dose = 16 mg/kg bodyweight), the tumor inhibition rate reached 56.2%, which was comparable to that of paclitaxel (57.3%) at a dose of 4 mg/kg bodyweight. Our results confirm that the structural modification of CX-PC-casein-NPs can effectively prolong the remaining time of specific drugs, and may provide a potential strategy for cancer treatment.

## 1. Introduction

Caseins have been widely studied and used due to their biocompatibility, biodegradability, bioresorbability, and low price, allowing for simple production. Casein proteins can form amorphous and stable agglomerate micelles—namely, casein micelles—under suitable conditions [1]. Moreover, caseins can enter through the plasma membrane in an energy-independent fashion, which may improve the cellular uptake of specific drugs [2]. Further, casein micelles show a porous inner structure [3,4] with irregular channels (*d* > 5 nm) and inner cavities (*d* = 20–30 nm) [3]. These features make caseins a promising matrix candidate for drug encapsulation [5,6]. The amphiphilic nature of caseins endows the nanoparticles a natural affinity for hydrophobic substances. However, casein nanoparticles loaded with hydrophobic drugs usually show low structural stability. Good stability was obtained only at 4 °C for 2 weeks for celecoxib (CX)-β-casein nanoparticles [7], for 34 days for docosahexaenoic acid (DHA)-casein nanoparticles [8], and for over one month for vitamin D_3_-casein nanoparticles [9]. When the drug-loaded casein nanoparticles were placed in the medium at 37 °C, most of the drugs, such as paclitaxel [10], celecoxib, and vitamin D (unpublished data), were quickly released within 5 min. This may result in unfavorable release profiles for specific drugs, as the bioavailability, targeting ability, and local toxicity should also be considered. Therefore, it is critical to enhance the physicochemical stability of drug-loaded casein nanoparticles for drug delivery in vivo via the structural modification of casein nanoparticles.

Celecoxib (CX) is clinically used for treating inflammation, such as arthritis, ankylosing spondylitis, and chronic pain. In addition, CX has been approved as a prophylactic for familial adenomatous polyposis [11]. Recently, there has been a rising interest in evaluating the efficacy of CX, either alone or in combined with other drugs, against several cancers in preclinical trials [12]. The inhibition of CX on caspase signaling has been reported as a molecular mechanism by preventing neoplastic progression and angiogenesis by decreasing COX-2-induced VEGF production [13,14,15,16,17]. Moreover, the anti-EMT (epithelial-mesenchymal transition) properties of CX have also been found by treating human colon and bladder cancer cell lines with CX [18,19]. However, clinical studies showed that oral COX-2 inhibitors at a higher dose raise cardiovascular risk [20].

Recently, celecoxib has been encapsulated in β-casein nanoparticles with no other additives. These CX-loaded nanoparticles have a high retention rate (10–20%, after freeze-drying), and can be resuspended without structural changes [8,21,22]. However, we found that CX-loaded β-casein nanoparticle dispersions were unstable at 37 °C, and more than 90% of the celecoxib leaked out of the nanoparticles within 5 min. 

In the present study, we performed a structural modification of casein nanoparticles using phosphatidylcholine (PC) to stabilize the loaded CX. The CX-PC-casein-NPs yielded a significant enhancement of anti-tumor activity when the nanoparticle dispersion was administered by intravenous injection in mice. 

## 2. Materials and Methods 

### 2.1. Materials

Sodium caseinate, celecoxib (CX), carbamazepine, bovine β-casein (>97%), EGTA, and HEPES buffer were purchased from Sigma-Aldrich (Shanghai, China). Paclitaxel (PT, >98%) and PBS (phosphate buffer saline) buffers with different pH values were purchased from Shanghai Yuanye Biotechnology Co., Ltd. (Shanghai, China). All materials for the cell culture were obtained from Sangon Biotech (Shanghai) Co., Ltd., China. Egg yellow phosphatidylcholine, with a purity of 98% (PC), was purchased from Shanghai Avt Pharmaceutical Technology Co., Ltd., Shanghai, China.

### 2.2. Preparation and Structural Modification of Casein Nanoparticles

All casein nanoparticles (NPs) were prepared with sodium caseinate (20 mg/mL) by magnetic-stirring for 30 min (500 rpm, 25 °C). After the stirring, 100 μL PC (100 mg/mL) in absolute ethanol was added dropwise (20 μL/min) to 20 mL of casein nanoparticle dispersion. After the addition of PC, the dispersion was treated using an ultrasonic (FS-350T Ultrasonic system, Shanghai Shengji Ultrasound Instrument Co., Ltd., China) for 5 min to perform the structural modification of the casein nanoparticles, after which the PC-casein nanoparticles were obtained.

To further understand the ability of PC to modify the casein nanoparticles, the PC solution (100 μL) was added dropwise (20 μL/min) to 20 mL water and treated for 5 min using ultrasonic. The particle sizes and microstructures were observed by a transmission electron microscope (TEM). 

The structural modification of the NPs was further analyzed using a SU9000 scanning electron microscope (SEM, Hitachi, Tokyo, Japan). The NP dispersion was snap-frozen using liquid nitrogen and then freeze-dried. The freeze-dried samples were then subjected to SEM. To obtain high resolution images, the SEM was performed under a high voltage of 30 kV.

### 2.3. Celecoxib Loading of PC-Casein Nanoparticles

Celecoxib (CX) was pre-dissolved in absolute ethanol (100 mg/mL). A given amount of the solution was dropwise added to the casein nanoparticle dispersion with stirring at 25 °C. The CX amount within the final dispersion was 2 mg/mL (i.e., the mass ratio of casein protein/CX = 10:1). Following the addition of the CX solution, the dispersions were continuously stirred for 30 min at 25 °C. After the stirring, PC in absolute ethanol (100 mg/mL), was added dropwise to the dispersions (20 μL/min) and then treated ultrasonically for 5 min to yield PC-CX-casein nanoparticles with modified structures. Different levels of PC were tested for structural optimization. 

### 2.4. Stability Study of PC-CX-Casein Nanoparticles

To confirm the stability of the casein nanoparticles, the dispersions were kept in sealed vials and stored at different temperatures (2–8 °C, 25 °C, or 37 °C). The changes of the particle size, polydispersity index (PDI), zeta potential (ZP), and entrapment efficiency (EE) of the dispersions were determined at predetermined time intervals [23]. In particular, EE studies of the CX in the nanoparticles were carried out via high-performance liquid chromatography (HPLC). After removing the free CX in dispersions with a 0.45 μm filter, the CX in the nanoparticles was extracted by methanol. Briefly, 50 μL of the dispersion was added to 450 μL of methanol to remove the protein by filtrating it with a 0.22 μm filter. The filtrate was then subjected to a CX analysis by HPLC. The HPLC analysis was carried out on a Shimadzu HPLC system composed of two LC-10A pumps, an SIL-10Avp autosampler, an SPD-M10Avp UV detector (Shimadzu, Kyoto, Japan), and a Symmetry C18 (5 m, 4.6 mm × 250 mm) column. The mobile phase for all experiments consisted of 30% water and 70% methanol. All of the samples were measured at 280 nm, with a flow rate of 1 mL/min. The column temperature was set at 30 °C. Since CX-casein nanoparticles could enter through a 0.45 μm filter but free CX could not, the loading amounts (mg/g) were calculated from the results of the HPLC analysis. The EE (%) of CX in the casein nanoparticles was calculated according to the following equation: EE (%) = 100 × (CX in casein nanoparticle dispersion)/CX in initial casein nanoparticle dispersion. 

### 2.5. Preparation of CX-β-Casein Nanoparticles

To evaluate the stability of the PC-CX-casein nanoparticles, bovine CX-β-casein nanoparticles were prepared according to a previous method [7]. Briefly, 20 mg/mL bovine β-casein (Sigma-Aldrich, Shanghai Branch, China) was dissolved in 20 mM HEPES buffer (pH 7.0, Sigma-Aldrich, Shanghai Branch) containing 1 mM MgC1_2_, 2 mM EGTA (Sigma-Aldrich, Shanghai Branch) and 10 mM NaCl and was stirred overnight at 4 °C. CX dissolved in absolute ethanol was added dropwise to the protein nanoparticle dispersion under stirring over 30 min at 25 °C. The CX-β-casein nanoparticles contained β-casein and CX with a mole ratio of l:8 (the mass rate of casein protein/CX = 10:1). The CX-β-casein nanoparticles are used as a reference for the characterization of the PC-CX-casein nanoparticles.

### 2.6. Characterization of Casein Nanoparticle Dispersions

The casein nanoparticle dispersions were analyzed using a Zetasizer ZSE (Malvern Instruments, Malvern, UK) to obtain information for the nanoparticles, including the size distribution, zeta potential (ZP), and polydispersity index (PDI). 

### 2.7. Cell Culture

Cells were cultured in DMEM (Gibco BRL, Grand Island, New York, USA). The culture medium contained 10% fetal bovine serum, 100 U/mL penicillin, and 100 µg/mL streptomycin (Sigma-Aldrich, Steinheim, Germany). The monolayer cells were cultured in 5% carbon dioxide, and the temperature was maintained at 37 °C.

### 2.8. Cell Viability Assay

An MTT assay was used to evaluate cell viability. Briefly, the cells were plated in a 96-well plate (5000 cells per well) and cultured for 24 h. When the cells reached a confluence of 60%, the drugs at the given doses were added into the wells and continued to culture for 48 h. After that, the medium was removed, and the cells were washed twice by PBS; then, 200 μL medium containing 5% FBS and 150 μL MTT reagent was added and incubated for 4 h at 37 °C. After the incubation, the medium was removed, and 150 μL of dimethyl sulfoxide (Shanghai Yuanye Biotechnology Co., Ltd., Shanghai, China) was added to each well. The well plate was then shaken to dissolve the purple formazan precipitate. The absorbance of each well was recorded by a Bio-Rad micro plate reader (Bio-Rad Life Medicine Products (Shanghai) Co., Ltd., China) at 490 nm. The inhibition rate of the drugs was calculated according to the optical density values. 

### 2.9. In Vitro Cell Uptake Studies

Cell uptake studies of CX were performed with human malignant melanoma cells (A375) according to a feasible procedure. Briefly, A375 cells (5 × 10^5^ cells/mL) were plated on 24-well cell culture plates (Nunclon Delta; Nunc, Wiesbaden, FRG) in an RPMI 1640 media containing 10% heat-inactivated fetal bovine serum (Gibco) and 1% antibiotics. After 6 h of incubation (37 °C/5% CO_2_), the non-adherent cells were separated, and the adherent cells were treated with CX, CX-β-casein NPs, and CX-PC-casein-NPs (Equivalent to CX 30 μg/mL in the final culture medium), reconstituted in RPMI 1640 medium and incubated for 24 h in the same conditions. At the end of 24 h, the culture medium and non-adherent cells were removed by centrifugation (10,000× *g*). The clear culture medium was extracted two times by isochoric ethyl acete. The extraction solution was evaporated for dryness, and the residue was dissolved in methanol (1.0 mL) for CX determination in the culture medium by ultraperformance liquid chromatography-tandem mass spectrometry (UPLC-MS/MS). Then, 5 mL 50% ethanol was added to the well to dissolve the precipitated CX and to extract the extracellular CX three times since the CX amount in the fourth extraction solution was negligible. The obtained extraction solution was subjected to UPLC-MS/MS for the determination of the extracellular CX and precipitated CX. After the extraction of extracellular CX and precipitated CX, the adherent cells were scraped using a cell scraper and washed repeatedly 3 times with 100% ethanol. The obtained cells plus the ethanol solution (about 10 mL) were treated with an ultrasonic cell pulverizer to extract the intracellular CX. The extraction solution and pulverized cell residue were separated by centrifugation (10,000× *g*), and the supernatant was subjected to UPLC-MS/MS to determine intracellular CX. The UPLC-MS/MS analysis was performed according to the method reported by Zheng et al. [24]. The UPLC system (Waters, Milford, MA, USA) equipped a binary solvent manager and a sample manager with a flow-through needle and a Waters XEVO TQD triple-quadrupole with an electrospray ionization source and a BEH C18 column (2.1 × 50 mm, 1.7 μm). The Masslynx 4.1 software (Waters Corporation, Milford, MA, USA) was used for data acquisition and the instrument control. The mobile phase was composed of 0.1% formic acid (A) and acetonitrile (B) with a gradient: 40% (A)−60% (B) during 0–0.5 min, 60% to 95% (B) during 0.5–1.5 min, 95% to 60% (B) during 1.5–2.0 min and 60% (B) during 2.0–2.5 min, at a flow rate of 0.4 mL/min. CX was analyzed using the multiple reaction monitoring (MRM) method with a positive ion mode. The cone voltage was set at 60 V, and the collision voltage was set at 40 V and 20 V for the ion mass spectrometric analysis of m/z 381.7–362.2 of CX, respectively.

### 2.10. Determination of Drug Concentration-Time Curve

Male Sprague-Dawley rats (10, 260–300 g) were divided into group A and group B (*n* = 5) randomly. A silastic catheter was inserted into the right jugular vein of each rat under anesthetic using pentobarbital (65 mg/kg ip.). The rats in group A received CX-β-casein-nanoparticles with a CX of 5 mg/kg b.w., and those in group B received CX-PC-casein-nanoparticles with a CX of 5 mg/kg b.w. via the caudal vein. At 0.083, 0.5, 1, 2, 4, 8, 12, and 24 h after drug administration, 300–400 µL blood samples were collected with a heparin-treated tube through a silastic catheter in the right jugular vein. The obtained blood samples were immediately treated by centrifugation (13,000 rpm, 10 min), and then the plasma was transferred into a clean tube (0.5 mL), which was kept at −80 °C for CX determination. 

The plasma (100 µL) was placed into a 1.5 mL tube, to which was added 20 µL carbamazepine (IS) (500 ng/mL) and 300 µL acetonitrile. This mixture was then mixed for 2 min using a vortex. The mixture were centrifuged for 10 min (13,000 rpm). The supernatant diluted with water (1:1) (2 µL) was injected into UPLC-MS/MS for CX analysis, which was described in the previous section. In the MRM method, the collision voltage was set at 40 V for the IS with an ion mass m/z of 237.1–194.2.

Winnonlin Professional Edition version 5.2 (Pharsight Corp, Cary, NC, USA) was employed to analyze the pharmacokinetic data with noncompartmental methods. The area under the concentration versus time curve (AUC) from 0 to *t* (AUC_0-*t*_) was calculated using the linear up/log down rule. The volume of distribution (*V*_d_) and clearance (CL) was calculated using standard equations. The maximum concentration (*C*_max_) and time of *C*_max_ (*T*_max_) were determined by visual inspection of the concentration versus the time curve data.

### 2.11. Tumor Inhibition Experiments In Vivo

Animal experiments (No. SYXK (Zhejiang) 2018-0008) were performed according to the relevant rules and ethical guidelines in Zhejiang Academy of Medical Sciences, China. Four-week-old HRS/J male hairless mice (50) (18–20 g) were subcutaneously injected with 0.2 mL A-375 human melanoma cells (8 × 10^9^/L) in the axilla of their left forelimb. The mice were then housed individually in plastic cages in an air-conditioned room (22 ± 2 °C, 55 ± 5% humidity), which was maintained on a 12-h light/12-h dark cycle. The mice were given free access to food and water. After 14 d, 24 mice reached a median tumor volume of 203 mm^3^. The mice were randomly divided into four groups labelled I–IV. Group I (the control group) was fed according to a normal schedule. The other groups were treated with the following formulations (once every 3 days): II, CX-PC-casein nanoparticle dispersion (8 mg/kg body weight (b.wt.) CX); III, CX-PC-casein dispersion (16 mg/kg b.wt. CX); IV, PT (4 mg/kg b.wt. PT). Drugs were administered via the caudal vein in a volume of 0.1 mL/10 g b.wt. The tumor volume (mm^3^) = (W^2^ × L)/2, where W is the width and L is the length (in mm) of the tumor, obtained by a caliper. Two days after the fifth drug treatment, the tumors were harvested, photographed, and weighed after the mice were euthanized by cervical dislocation. 

### 2.12. Statistical Analysis

In this study, all experiments were repeated in triplicate, and the data are presented as the mean ± standard deviation. The SAS system for windows V8 was used for data analysis. Significant differences between the two groups were evaluated by a Student’s t test. Multiple comparisons were done by the least significant difference test. A probability value was considered to be significant or extremely significant at *P* < 0.05 or *P* < 0.01, respectively.

## 3. Results and Discussion

### 3.1. Structural Modification of Casein Nanoparticles by PC

**Figure 1:**
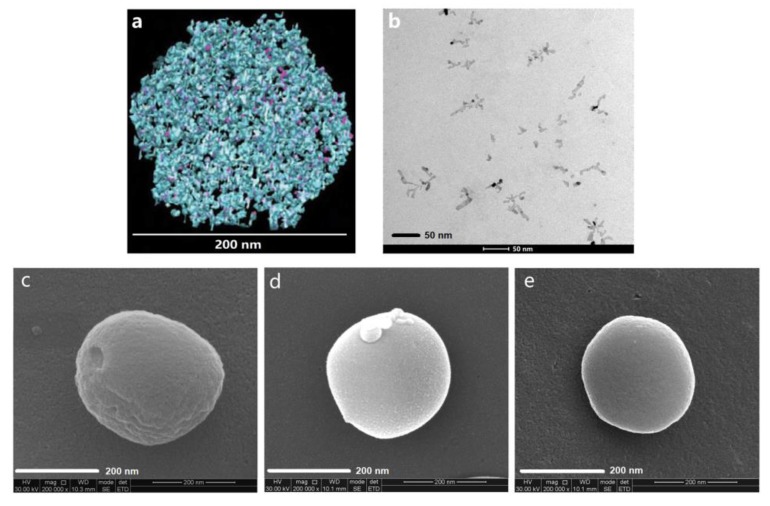
Modification of the casein-nanoparticles through phosphatidylcholine. (**a**) The image the casein-nanoparticle derived from cryo-transmission electron tomography comes from the literature [4], with permission from Elsevier, 2011; (**b**) TEM image of phosphatidylcholine (PC); (**c**) SEM image of casein-nanoparticles; (**d**) SEM image of PC-casein nanoparticles, (**e**) SEM image of CX-PC-casein-NPs.

Images (Figure 1a) obtained by cryo-transmission electron microscopy show that irregular channels (*d* > 5 nm) and inner cavities (*d* = 20–30 nm) commonly exist within the nanoparticles [4], which provide the space for drug loading. Lipophilic drugs, such as celecoxib (CX), can enter the channels and cavities through the gaps on the nanoparticles under stirring and ultrasonic irradiation conditions. Nevertheless, only part of CX can be kept in the channels and cavities through the hydrophobic interactions between hydrophobic amino acid residues and CX. Our results show that most of the CX leaked out easily through the gaps and spread over the nanoparticles, especially when the samples were heated to around 37 °C, which is similar to body temperature. Under these conditions, the bioaccessibility and bioavailability of CX can hardly be ensured. Therefore, the structural modification of casein nanoparticles is crucial to stabilize the drugs in casein nanoparticles. As we know, phospholipid molecules can form bilayer membranes with a thickness of about 3–6 nm [25,26,27]. Using the same ultrasonic treatment as that for the preparation of CX-casein nanoparticles, phosphatidylcholine (PC) in water can form membranes, which may block the gaps in the casein nanoparticles (Figure 1b). Thus, we performed a structural modification of casein nanoparticles by phosphatidylcholine (PC). The obtained PC-casein-nanoparticle dispersion was snap-frozen using liquid nitrogen and then freeze-dried to yield well-structured nanoparticles for scanning electron microscopy (SEM). Under a high voltage of 30 kV, the SEM image of the casein-nanoparticle without PC modification exhibited wrinkles and hollow structures (Figure 1c), while the PC-modified casein-nanoparticles showed a more properly sealed and compact surface (Figure 1d). After the CX was loaded into the casein-nanoparticles and modified with PC, the CX-PC-casein-NPs also showed a compact surface (Figure 1e). The modification site of the PC was mainly on the surface of the nanoparticles. Meanwhile, we found that the PC-casein nanoparticles only loaded a small amount of CX since PC modification prevented CX from entering into the modified nanoparticles. Thus, it is crucial to determine a proper drug-loading procedure considering the addition sequences of the different building blocks for PC-casein nanoparticles. Moreover, PC modification and CX-loading led to a slightly smaller size of nanoparticles (PC-casein nanoparticles and CX-PC-casein-NPs), which might be a result of the interaction between the ester chain of the PC/CX and the non-polar amino acid residues and between the cholinesteryl phosphate group of PC and polar amino acid residues.

### 3.2. The Stability of PC-CX-Casein Nanoparticles

**Figure 2:**
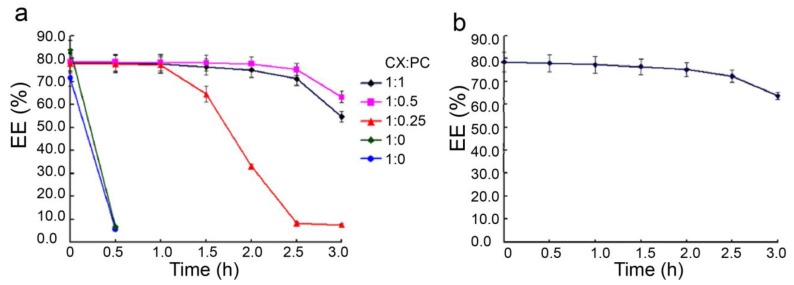
Stability of CX-β-casein-NPs (green) and CX-casein-nanoparticles (blue, red, black, and purple), indicated by the change of the entrapment efficiency (%) of CX in the nanoparticles at 37 °C. (**a**) Native nanoparticles (pH 6.8), (**b**) CX-PC-casein-nanoparticles with a 1:0.5 ratio of CX/PC at pH 7.4.

Considering the actual implemented temperature of intravenous injection, the stability of the CX-PC-casein-NPs was determined at 37 °C. Our previous study showed that CX-β-casein nanoparticles with casein protein/CX = 10:1 could only be stabilized for 2 weeks at 4 °C [7]. The CX-PC-casein-NPs made of 20 mg/mL casein protein contained about 80% CX when the amount of CX was 10% the amount of caseins, which is similar to the results for CX-β-casein nanoparticles. When PC was introduced to the nano-based delivery system, the obtained CX-casein nanoparticles were relatively stable at 37 °C, while the CX-β-casein nanoparticles stabilized in less than 5 min (green line) according to an unaided eye observation. CX-PC-casein-nanoparticles with PC 1/4, 1/2, and 1/1 CX content remained stable for 1, 2.5, and 2 h, respectively (Figure 2a). When half the CX amount of PC was added, the nanoparticles showed the best stability. Moreover, the stability of the CX-PC-casein-nanoparticles with a PC/CX ratio of 1/2 at pH 7.4 (37 °C) was very similar to that at pH 6.8 (37 °C)—the pH of the fresh dispersion, indicating that the nanoparticles were stable at pH 6.8–7.4. This pH range covers the pH of human body fluid (Figure 2b).

### 3.3. Resuspension of the Nanoparticles after Lyophilization

We measured the structural characteristics of the CX-casein nanoparticles and CX-PC-casein-NPs in fresh nanoparticle dispersions and made a comparison with the nanoparticle dispersions reconstituted from the corresponding lyophilized powders, as β-casein nanoparticles can be resuspended and maintain their structural characteristics [7]. From the experimental results (Table 1), we can see that the mean diameter, polydispersity index (PDI), and zeta potential of the lyophilized casein-nanoparticles were approximately the same as those of the pre-lyophilized CX-PC-casein-NPs. However, the lyophilized CX-casein nanoparticles failed to resuspend, as CX leaked out from the nanoparticles after lyophilization, which means that PC plays an important role in maintaining the microstructure of the nanoparticles. The re-dispersion of the CX-PC-casein-NPs also remained stable for 2.5 h at pH 7.4.

### 3.4. Inhibition on Tumor Cells In Vitro

**Figure 3:**
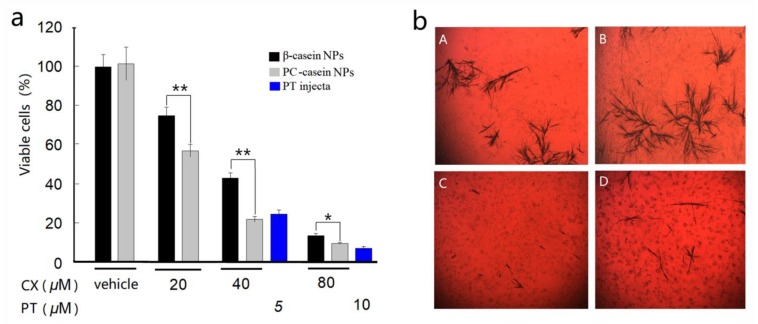
Effects of the two CX-casein nanoparticles (NPs) with different stability at 37 °C on A375 cell viability compared to paclitaxel (PT). (**a**) Cell viability at different CX concentrations, * *P* < 0.05, ** *P* < 0.01; (**b**) crystalline CX observations after 48 h when the CX-casein nanoparticles were added to the cell culture medium. (A) CX-PC-casein-NPs with 80 μM CX, (B) CX-β-casein-NPs with 80 μM CX, (C) CX-PC-casein-NPs with 40 μM CX, and (D) CX-β-casein-NPs with 40 μM CX.

In this study, the effects of the CX-casein nanoparticle dispersions on the cell viability of human malignant melanoma cells (A375) were investigated compared to those of paclitaxel (PT). Morphological changes were observed in A375 human melanoma cells in response to celecoxib nanoparticle formulations. Untreated A375 cells were adherent and serried, showing a normal triangular or spindle morphology. After exposure to celecoxib, cell adherence and density were significantly decreased. Spindle cells became round, and a large number of floating cells were observed. The results show that the relatively stable CX-PC-casein-NPs had higher efficiency in inhibiting A375 cells than the unstable CX-β-casein-NPs at 37 °C (Figure 3a). When the CX concentration reached 40 μM (15.2 μg/mL), the inhibition efficiency (calculated according to the % of viable cells) of the CX-PC-casein-NPs was 76.6%, which was higher than that of 5 μM PT (4.3 μg/mL). At 80 μM CX, the nanoparticles showed a high efficiency in inhibiting A375 cells (>85%). Nevertheless, when the drug concentration was increased to 80 μM, the inhibition rates of the CX-loaded micelles were significantly lower than those of 10 μM PT (92%). Within the cell culture medium treated with 40 μM CX samples, we observed different amounts of crystalline CX deposited on the bottoms of the wells (Figure 3b). The crystalline form of CX might be a critical factor affecting bioaccessibility, bioavailability, and, consequently, the cell inhibition rate of CX-loaded nanoparticles. In particular, the relatively stable CX-PC-casein-NPs were able to provide more bioavailable CX to inhibit A375 cells than the other NPs. However, both the relatively stable CX-PC-casein-NPs and unstable CX-β-casein-NPs at 37 °C yielded a high inhibition rate of A375 cells at a CX level of 80 μM. It is possible that too much CX was added to the culture system, most of which was deposited at the bottom of the wells, directly interacted with the cells, and caused the death of the adherent A375 cells (Figure 3b).

### 3.5. In Vitro Cell Uptake of CX

**Figure 4:**
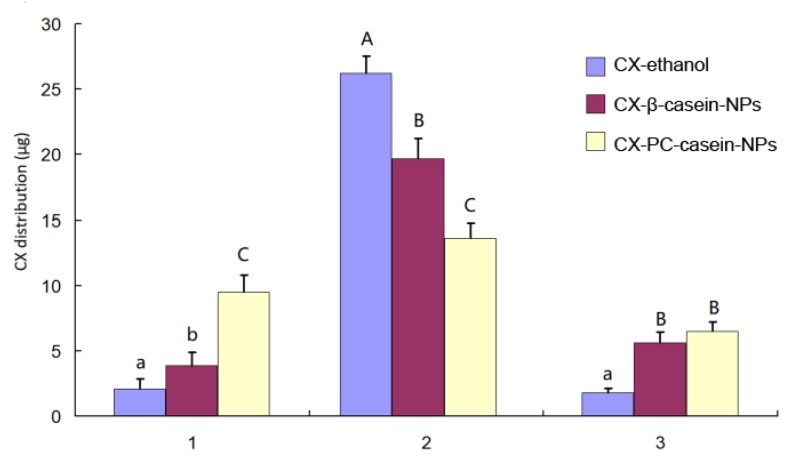
The CX distribution in the intracells (1) as crystallization, in the extracells (2), and in the culture medium (3) after the 24 h culture when 30 μg CX was added into each well containing 1 mL of the culture medium. Different lowercase letters over the bars in each group indicate significant differences at *P* < 0.05, and a lowercase letter and a different capital letter, or different capital letters, indicate significant difference at *P* < 0.01.

Drug uptake by cancer cells is critical to the growth inhibition on the cells. To evaluate the effect of CX-PC-casein-NPs on cellular uptake, 375 cells were used in the study. In the present study, the cells were treated with CX-ethanol, CX-β-casein-NPs, or CX-PC-casein-NPs with CX 30 µg/mL in each well, and the extent of cell uptake is estimated by measuring the CX amount precipitated in the well bottom plus the extracells, culture medium, and intracells. The experimental data are given in Figure 4. The results show that a significant increase (*P < 0.05*) in cellular uptake occurs for CX-PC-casein-NPs compared to CX-β-casein-NPs, as well as for CX-β-casein-NPs compared to CX-ethanol. The increased cellular uptake in CX-PC-casein-NPs was 2.4 times that of CX-β-casein-NPs and 4.5 times that of CX-ethanol. More stable nanoparticles resulted in a more enhanced cellular uptake of CX. Correspondingly, the CX amount precipitated in the well bottom, and extracells showed a contrary result that was consistent with the observation in the inhibition test of the tumor cells in vitro. In the culture medium, the CX-ethanol group exhibited a CX concentration of 1.8 µg/mL, while the CX-β-casein-NPs and CX-PC-casein-NPs groups yielded CX concentrations of 5.6 and 6.5 µg/mL since the casein proteins in the culture medium retained some CX.

### 3.6. Pharmacokinetics of NP Formulations with Different Stability

**Figure 5:**
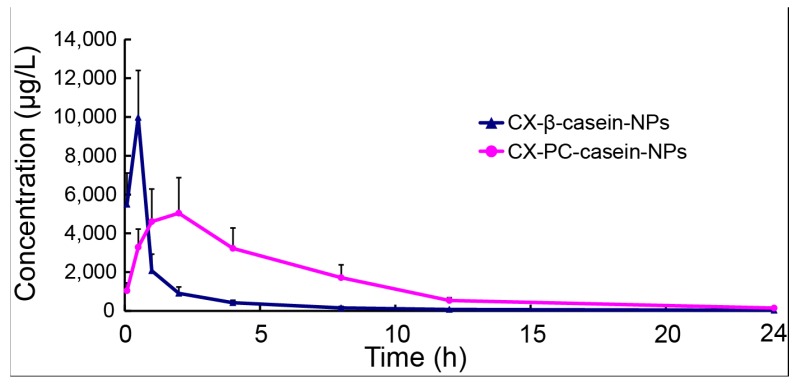
Concentration–time curve following 5 mg/kg CX b.wt. with two nano-formulations, administered via the caudal vein (*n* = 5). Data are presented as the mean ± standard deviation.

To further understand the importance of the stability of casein nanoparticles administrated by intravenous administration, the CX pharmacokinetics of CX-PC-casein-NPs and CX-β-casein-NPs were analyzed based on a drug concentration–time curve. After the single intravenous administration of CX-PC-casein-NPs or CX-β-casein-NPs at a dosage of 5 mg CX/kg CX b.w., the plasma concentration profile was obtained (Figure 5). This profile showed that the CX-PC-casein-NPs maintained drug concentrations above 2 μg/mL in vivo for about 8 h, but only for 1 h for the CX-β-casein-NPs. Obviously, the CX-PC-casein-NPs showed advantageous sustained release properties. Table 2 shows the major pharmacokinetic parameters of CX after intravenous drug injection. The AUC of the concentration–time curve of the CX-PC-casein-NPs was 2.9-fold higher in rats compared with that of the CX-β-casein-NPs. The *C*_max_ of the CX-PC-casein-NPs was much less than that of the CX-β-casein-NPs, while the clearance (CL) and the volume of distribution (*V*_d_) of the CX-PC-casein-NPs were also much less than those of the CX-β-casein-NPs. These results demonstrate that CX delivery using the stable nanoparticles was more efficient than that using the unstable nanoparticles.

### 3.7. The Inhibition Effect of CX-PC-casein-NPs on Tumor Growth

To further verify the inhibitory effects of CX-casein-nanoparticles with different stability *in vivo*, we sought to inject the dispersions of CX-β-casein-NPs and CX-PC-casein-NPs via the caudal vein every 3 days. Before the third injection, we found that the CX-β-casein-NPs group showed severe red swelling or ulcerations at the injection sites of the tails. However, no ulceration at the injection site of the tails was found in the CX-PC-casein nanoparticle group. As we know, CX can cause gastrointestinal ulcerations after oral administration. Thus, the red swelling or ulcerations at the injection site were likely caused by the high CX concentrations due to CX leakage from the casein nanoparticles at body temperature. The improved stability of CX-PC-casein-NPs eliminated the local high CX concentrations though CX-PC-casein-NPs, which were stable for 2–3 h at body temperature. This also suggests that CX-PC-casein-NPs could be relatively stable in the blood environment.

**Figure 6:**
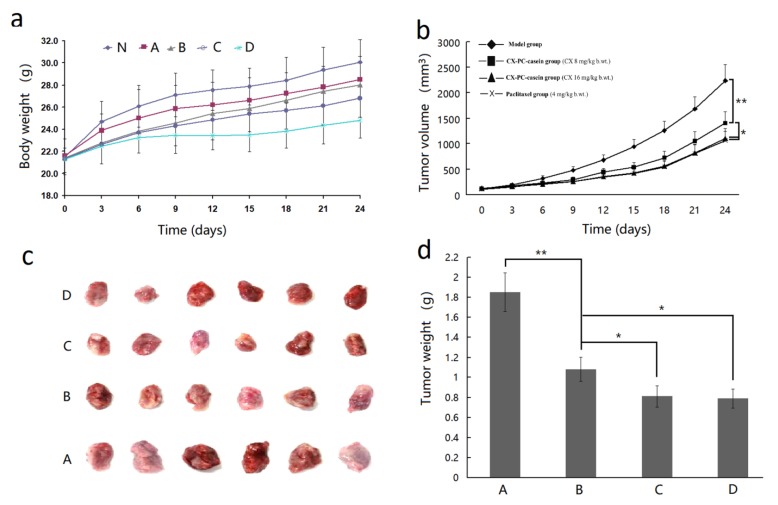
Results of animal experiments with different drug treatments. (**a**) Bodyweight changes, (N) Normal group, (A) model group, (B) CX-PC-casein nanoparticle group (CX 8 mg/kg b.wt.), (C) CX-PC-casein group (CX 16 mg/kg b.wt.), and (D) paclitaxel group (4 mg/kg b.wt.); (**b**) changes in tumor volume; (**c**) tumors after the final drug treatment; (**d**) tumor weight after the final drug treatment. * *P* < 0.05, ** *P* < 0.01.

Mice implanted with A375 tumors were administered the formulation of CX-PC-casein-NPs via the caudal vein every 3 days for 24 consecutive days. The body weight (b.wt.) and tumor volumes of the mice were then measured. All mice exhibited normal behavior, and there were no fatalities. The changes in body weight are shown in Figure 6a. The results indicate that a high dosage of CX-PC-casein-NPs (16 mg/kg b.wt. of CX) notably decreased bodyweight. However, the effect of CX-PC-casein-NPs was less negative than that of PT (4 mg/kg b.wt.) (*P* < 0.01). This indicates that CX-PC-casein-NPs had low toxicity for the mice. Compared with the control group, the treatment groups administered with CX-PC-casein-NPs containing both 8 and 16 mg CX/kg b.wt. showed a significant inhibition of the growth of the implanted A375 tumors (Figure 6b–d). The final tumor inhibition rate was 41.6% for the mice supplemented with CX-PC-casein-NPs (8 mg CX/kg b.wt.). In contrast, the tumor inhibition rate of the CX-PC-casein-NPs reached 56.2% for the mice treated with 16 mg CX/kg b.wt. A similar inhibition efficacy was observed among the animals administered with 4 mg/kg b.wt. PT (57.3%). These data clearly indicate that CX-PC-casein-NPs have the potential to treat tumor growth. Malignant melanoma is the most aggressive skin cancer, with fast metastasis, high mortality, and poor prognosis [28]. We, therefore, believe that CX-PC-casein-NPs show potential as a formulation for the intravenous administration of CX to treat cancer, since PC and caseins are safe for patients.

CX is commercially used in many countries and is commonly administered two or three times (200 mg/time) a day to treat arthritis, menstrual pain, and chronic pain [29]. However, it exhibits poor solubility and variable absorption, which can lead to severe toxicity [14]. Although the present study focuses on intravenous drug delivery, we believe that the CX-PC-casein-NPs, which are composed of PC and caseins that exist in mammals, also provide a promising alternative oral formulation of CX. Compared with β-casein nanoparticles, CX-PC-casein-NPs are low-cost and more stable for practical use.

Recently, nano-medicines have been studied extensively. However, the stability of the nano-formulation of these drugs is a critical problem [30]. In this study, PC was used to improve the stability of CX-casein nanoparticles in their dispersions for the first time. This strategy may apply to the further development of stable casein nanoparticles for loading other hydrophobic drugs in the pharmaceutical industry.

## 4. Conclusions

The structural modification of casein nanoparticles through phosphatidylcholine to stabilize the loaded drug was successfully achieved. SEM shows that phosphatidylcholine (PC) blocks gaps in the nanoparticles to improve nanoparticle stability. CX-PC-casein-nanoparticles (196 nm) with a 1/2 ratio of PC/CX remained stable for 2.5 h under intestinal pH conditions at 37 °C. The freeze-dried nanoparticles could be resuspended with a mean size of 202.4 nm, without the loss of stability. The stability of CX-casein nanoparticles was found to be positively correlated with their inhibition activity against human malignant melanoma A375 cells and to eliminate the local high CX concentration at the injection site. The more stable CX-PC-casein-NPs resulted in 2.4 times the CX intracellular uptake than that of the unstable CX-β-casein NPs. A pharmacokinetic study showed that the AUC of the CX-PC-casein-NPs was 2.9-fold higher in rats compared to that of the CX-β-casein NPs. The CX-PC-casein-NPs with a CX dose of 16 mg/kg body weight, intravenously administered to mice implanted with A375 tumors, showed a similar tumor inhibition rate to paclitaxel (PT) at a dose of 4 mg/kg body weight. These CX-PC-casein-NPs, therefore, have potential for cancer treatment. For the first time, in this study, PC was used to improve the stability of the CX-casein nanoparticles in their dispersions. This strategy for the structural modification of casein nanoparticles using PC should also be effective for loading other hydrophobic drugs.

## Figures and Tables

**Table 1:** Characterization of different casein-CX nanoparticles.

Nanoparticles.	Mean Diameter (nm)	Polydispersity Index (PDI)	Zeta Potential (mV)	CX in Dispersion (mg/mL)
Fresh nanoparticle dispersion				
CX-casein nanoparticles	254.4 ± 5.4	0.340 ± 0.29	−44.57 ± 2.11	7.3
CX-PC-casein-NPs	192.6 ± 4.3	0.175 ± 0.09	−37.21 ± 1.47	8.5
Reconstructed nanoparticle dispersion from the corresponding lyophilized powder				
CX-casein nanoparticles	/^a^	/	/	/
CX-PC-casein-NPs	202.4 ± 2.9	0.188 ± 0.18	-36.87 ± 1.77	8.4

^a^ undetermined because precipitate yielded due to the leakage of CX.

**Table 2:** Pharmacokinetic parameters for CX-β-casein-NPs and CX-PC-casein-NPs.

Parameter	Formulation	
	CX-β-casein-NPs	CX-PC-casein-NPs
AUC_(0-24)_ (μg/L h)	11882.6 ± 1545.6	34574.3 ± 7844.9
t_1/2_ (h)	2.91 ± 0.40	4.51 ± 0.37
C_max_ (μg/L)	9974.2 ± 2426.3	6165.8 ± 1063.5
T_max_ (h)	0.5 ± 0	1.6 ± 0.5
CL (L/h/kg)	0.42 ± 0.06	0.15 ± 0.04
Vd (L/kg)	3.1 ± 1.78	0.97 ± 0.29
